# Clinical trials of new drugs for Alzheimer disease

**DOI:** 10.1186/s12929-019-0609-7

**Published:** 2020-01-06

**Authors:** Li-Kai Huang, Shu-Ping Chao, Chaur-Jong Hu

**Affiliations:** 1 Dementia Center, Department of Neurology, Shuang Ho Hospital, Taipei Medical University, New Taipei City, Taiwan; 2 The PhD Program for Neural Regenerative Medicine, College of Medical Science and Technology, Taipei Medical University, National Health Research Institute, Taipei, Taiwan; 3 Graduate Institute of Biomedical Informatics, College of Medical Science and Technology, Taipei Medical University, Taipei, Taiwan; 4 Neurology, School of Medicine, College of Medicine, Taipei Medical University, Taipei, Taiwan; 5 Taipei Neuroscience Institute, Taipei Medical University, Taipei, Taiwan

**Keywords:** Alzheimer disease, Clinical trials of drugs, Neuroinflammation, Neuroprotection, Anti-amyloid, Anti-tau, Cognitive enhancement

## Abstract

Alzheimer disease (AD) accounts for 60–70% of dementia cases. Given the seriousness of the disease and continual increase in patient numbers, developing effective therapies to treat AD has become urgent. Presently, the drugs available for AD treatment, including cholinesterase inhibitors and an antagonist of the N-methyl-D-aspartate receptor, can only inhibit dementia symptoms for a limited period of time but cannot stop or reverse disease progression. On the basis of the amyloid hypothesis, many global drug companies have conducted many clinical trials on amyloid clearing therapy but without success. Thus, the amyloid hypothesis may not be completely feasible. The number of anti-amyloid trials decreased in 2019, which might be a turning point. An in-depth and comprehensive understanding of the contribution of amyloid beta and other factors of AD is crucial for developing novel pharmacotherapies.

In ongoing clinical trials, researchers have developed and are testing several possible interventions aimed at various targets, including anti-amyloid and anti-tau interventions, neurotransmitter modification, anti-neuroinflammation and neuroprotection interventions, and cognitive enhancement, and interventions to relieve behavioral psychological symptoms. In this article, we present the current state of clinical trials for AD at clinicaltrials.gov. We reviewed the underlying mechanisms of these trials, tried to understand the reason why prior clinical trials failed, and analyzed the future trend of AD clinical trials.

## Introduction

The World Alzheimer Report 2015 revealed that 46.8 million people worldwide were living with dementia in 2015, and the total global societal cost of dementia was estimated to be US $818 billion. Alzheimer disease (AD) is the most common dementia type and may account for 60–70% of dementia cases [1]. AD typically presents as progressive memory decline initially, which is accompanied or followed by other cognitive dysfunctions, such as visuospatial abnormalities, navigation difficulties, executive problems, and language disturbance. These cognitive impairments further affect daily life activities, and many behavioral psychological symptoms of dementia (BPSD) usually occur during the disease course.

Pathological evidence regarding AD shows that degeneration in cholinergic neuron–rich regions, namely the nucleus basalis of Meynert, frontal cortex, anterior cingulate cortex, and posterior cingulate cortex [2, 3], is associated with memory loss, agitation, and apathy. Acetylcholine (ACh) has been shown to be highly correlated with memory function, including memory encoding, consolidation storage, and the retrieval process [4–6]. Currently, at least three cholinesterase inhibitors (AChEIs) approved by the US Food and Drug Administration (FDA) are being used to treat AD, with some clinical improvement in cognition and global function [7]. However, AChEIs can only improve cognitive symptoms of AD for a certain period but cannot modify the disease course.

The real causes of AD are still unclear. Two pathological hallmarks of AD exist, in terms of senile plaques, which consist of amyloid fibrils composed of the amyloid-beta (Aβ) peptide and neurofibrillary tangles consisting of hyperphosphorylated tau protein [8–10]. Another essential finding is brain atrophy, particularly in the hippocampus [11]. The proposition that Aβ accumulation is the central event in AD pathogenesis was initially proposed by three independent groups in 1991 [12–14]. All the mutant genes of hereditary, autosomal, and dominant familial AD, including amyloid precursor protein (APP), presenilin 1, and presenilin 2, encode the major proteins involved in amyloid metabolism [14–16]. Patients with trisomy 21 have APP gene locations with more amyloid accumulation and high AD risk in late life because they have one more copy of the APP gene, which results in increased amyloid production [17]. Previous studies have shown that the cerebral deposition of Aβ fibrils can occur decades before an individual shows clinical symptoms [18]. Molecular imaging studies such as those using amyloid positron emission tomography (PET) have shown that Aβ deposition reaches a plateau before brain atrophy can be identified from structural magnetic resonance imaging (MRI) and cognitive symptoms [15, 19]. The amyloid hypothesis has been the mainstream explanation for AD pathogenesis for decades, but all the prior clinical trials involving amyloid burden reduction failed (Tables 1and 2).

**Table 1:** Failed phase 3 trials on anti-amyloid therapy in AD since 2016

Year	Drug	Mechanism of action	Participants	Main reasons for failure	Remarks
2016	Solanezumab	Monoclonal antibody	Mild AD	Lack of efficacy	
	Solanezumab	Monoclonal antibody	Prodromal AD	Strategic	
	Verubecestat	BACE inhibitor	Mild to moderate AD	Lack of efficacy	
2018	Verubecestat	BACE inhibitor	Prodromal AD	Lack of efficacy	Worsens cognition
	Atabecestat	BACE inhibitor	Preclinical AD	Toxicity	Worsens cognition
	Lanabecestat	BACE inhibitor	Early AD	Lack of efficacy	Worsens cognition
	Lanabecestat	BACE inhibitor	Mild AD	Lack of efficacy	Worsens cognition
2019	Aducanumab	Monoclonal antibody	Early AD	Lack of efficacy	
	CNP520	BACE inhibitor	Preclinical AD	Lack of efficacy	Worsens cognition

Tau accumulation, which might be a consequence of neuronal damage, was proposed to begin between AD clinical symptom development and Aβ accumulation [20]. Neurofibrillary tangles and quantitative neuronal loss, but not amyloid plaques, have been found to correlate with disease severity and dementia duration [21–23]. Moreover, PET studies have shown that the spatial patterns of tau tracer binding are closely linked to neurodegeneration patterns and the clinical presentation in patients with AD [24]. Recently, biomarkers of amyloid, tau, and neurodegeneration were used for precisely diagnosing AD [25].

**Fig. 1:**
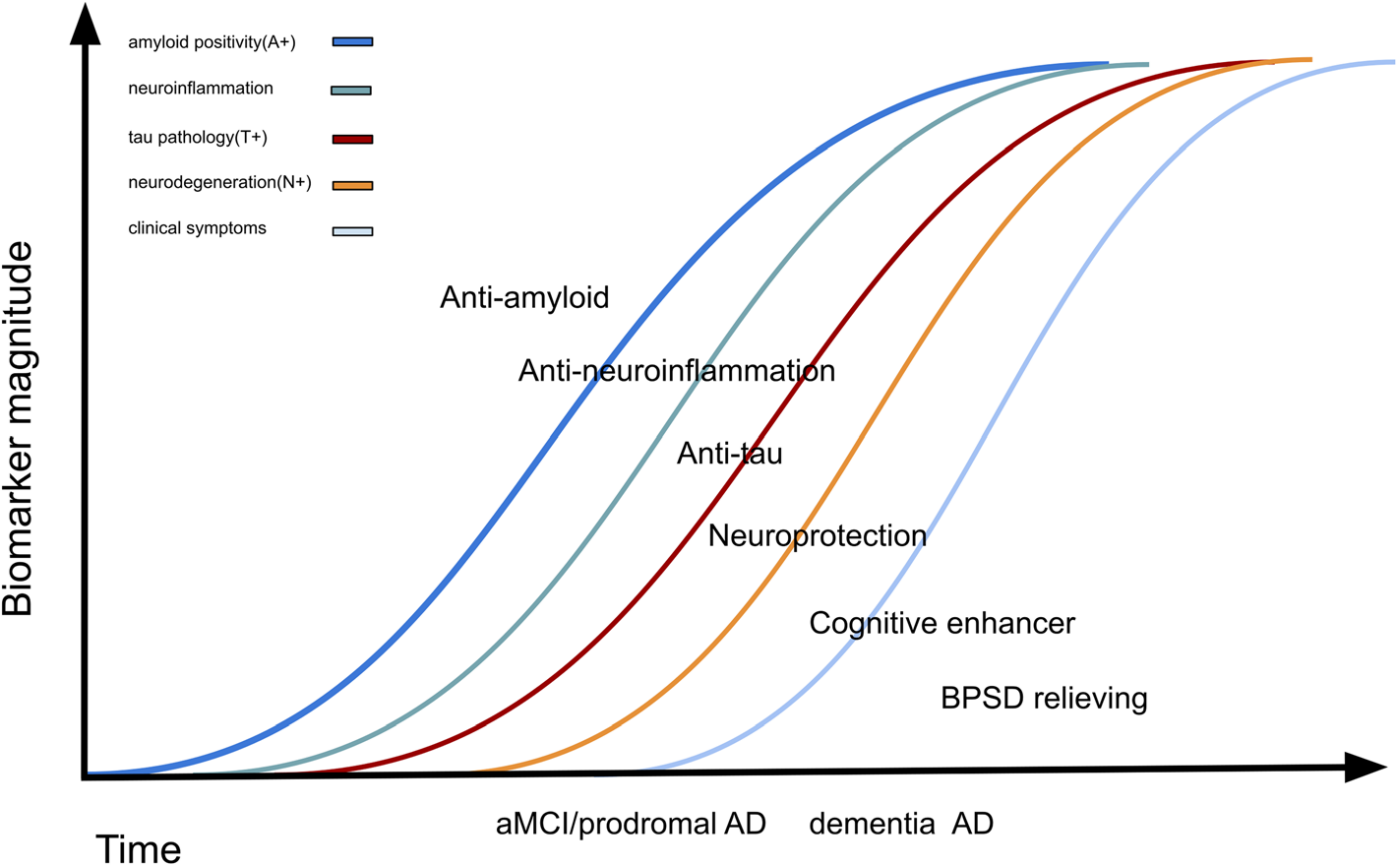
On the basis of the amyloid hypothesis, the consequent events of pathophysiology and clinical course are amyloid accumulation, neuroinflammation, tau accumulation, brain metabolism dysfunction, brain atrophy, cognitive decline (from mild cognitive impairment to dementia), and dementia symptom development. New drugs should target at least one of these events

**Fig. 2:**
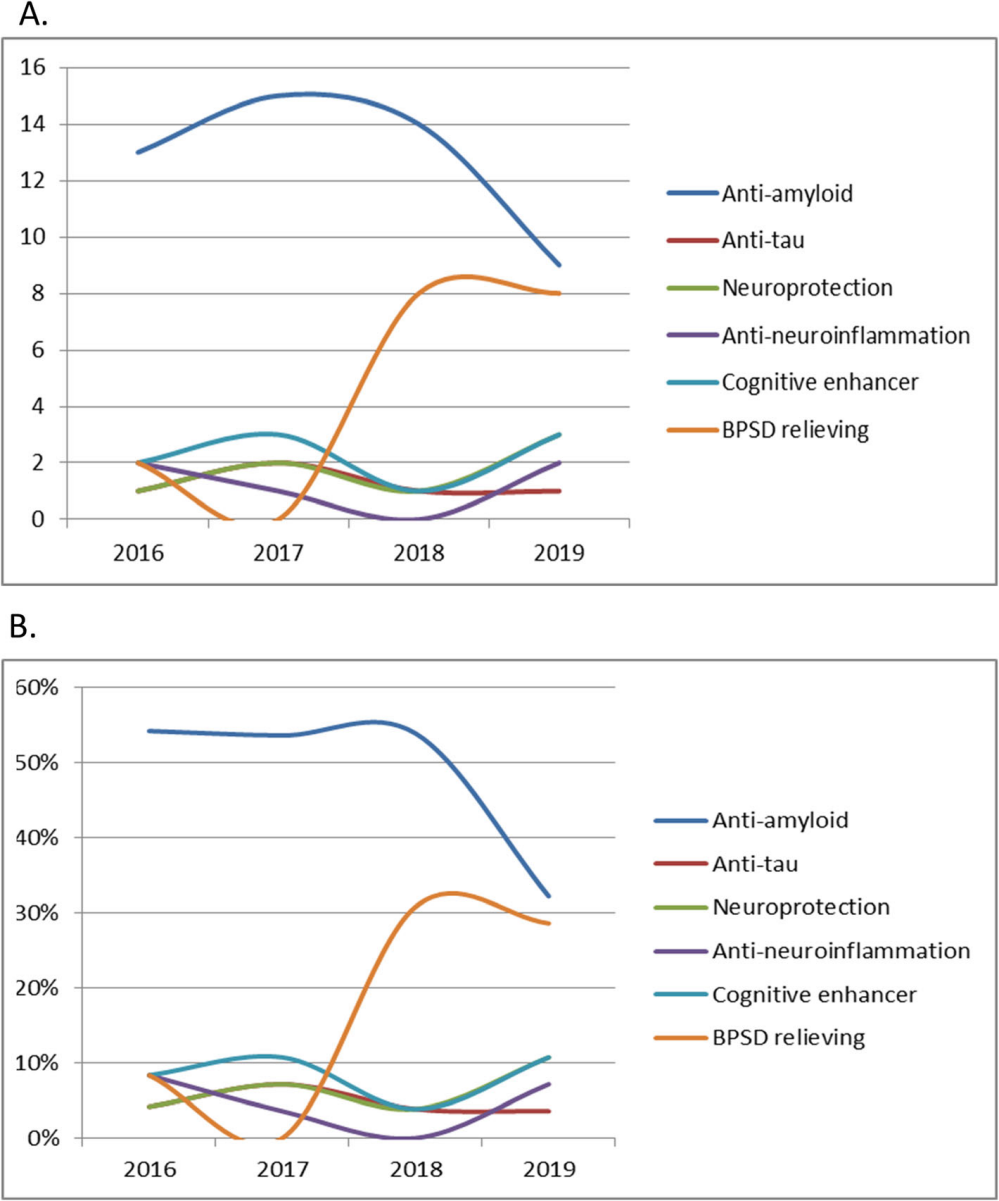
Trend of phase 3 trials, 2017–2019, according to the event-related categories at ClincalTrials.gov. **a** Number of phase 3 trials. **b** Percentage of phase 3 trials

**Fig. 3:**
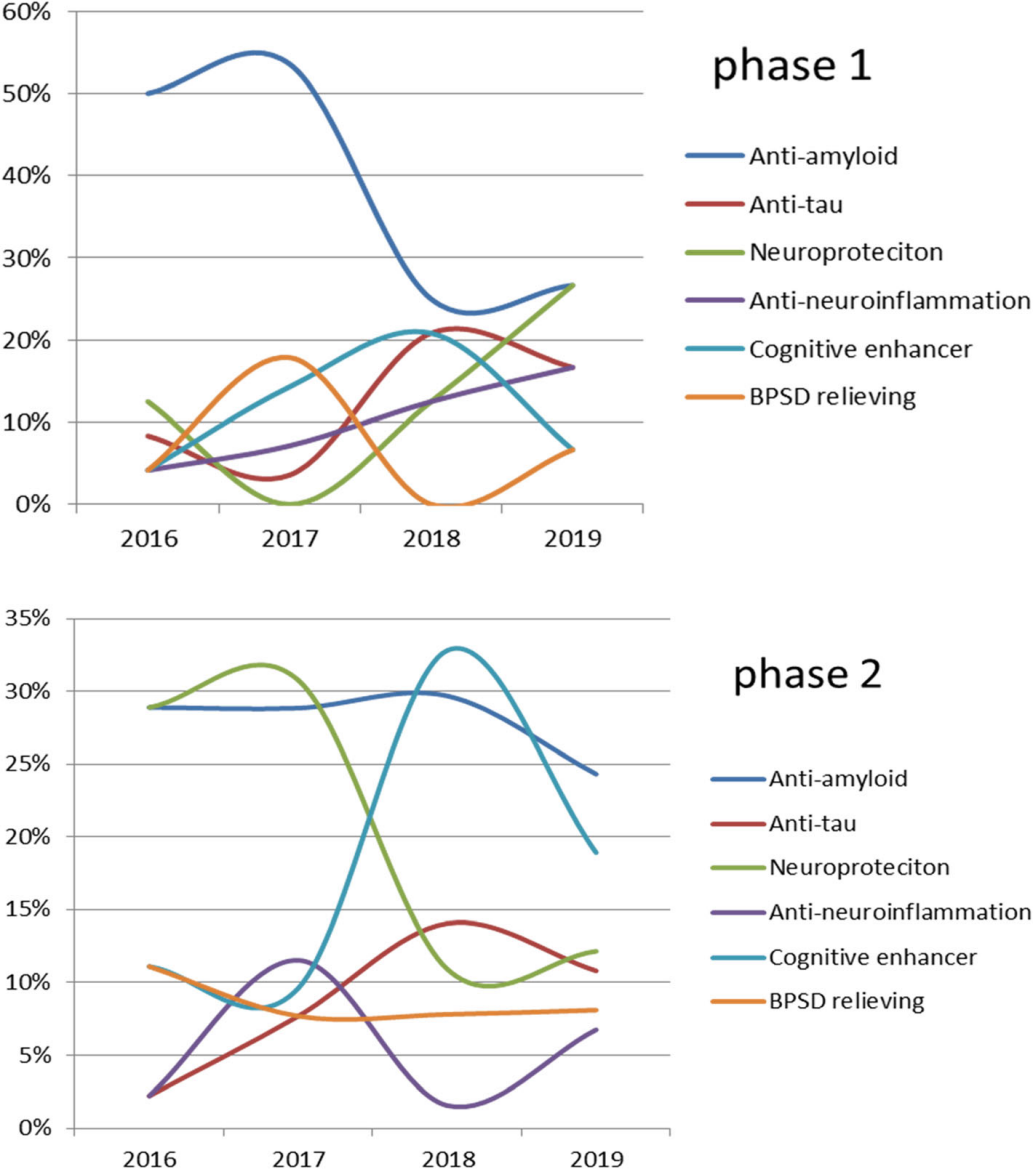
Trends of phase 1 and phase 2 trials, 2017–2019, according to the event-related categories in ClincalTrials.gov. The percentages of phase 1 and phase 2 trials

Furthermore, the brains of patients with AD exhibited evidence of sustained inflammation. Aβ itself acts as a proinflammatory agent, activating many inflammatory components. In the early stages of AD, initial microglial activation may serve a protective role (anti-neuroinflammatory), whereby it tries to clear the amyloid and release nerve growth factors. However, when Aβ or other toxic products over-accumulate, proinflammatory phenotypes are activated, which damage the neurons [26]. Moreover, the inflammatory response has been observed in many studies of postmortem tissues of patients with AD [27, 28]. Neuronal death or brain atrophy induced by amyloid, tau, and neuroinflammation might be prevented with neuroprotective therapies, which include suppressing excitable amino acid signaling pathways, free radical scavengers, and regeneration enhancers (Table 3) [29]. In addition to potentially disease-modifying therapies, many clinical trials focusing on symptomatic treatment, including enhancing cognitive functions and relieving BPSD, are ongoing (Table 3). In summary, molecular and clinical events occur subsequently in the disease course of AD. All such events are targets of the ongoing clinical trials of interventions for different AD stages (Fig. 1). The number of phase 3 trials for anti-amyloid therapy decreased in 2019 (Fig. 2). The lists of early-phase trials show a diverse trend (Fig. 3).

### Anti-amyloid therapy

A few approaches reduce the amyloid burden have been developed. Aβ is produced from APP, which is digested by gamma-secretase and beta-secretase [30–32]. Both gamma-secretase and beta-secretase inhibitors have been the targets of new drug development [33, 34]. Aβ is degraded by a few enzymes, including neprilysin, and has also been considered for new drug development [35, 36]. Removing Aβ through immunotherapy is also a reasonable strategy.

In 2019, nine phase 3 trials for eight drugs targeting amyloid are underway. Two of these enrolled patients with preclinical AD; one trial required positive amyloid PET, and the other required genetic mutation or strong genetic risks. Four trials enrolled patients with prodromal AD with positive biomarkers, with one trial for prodromal and mild AD and two for mild to moderate stages of AD-related dementia. The inclusion criteria for these trials were positive amyloid PET or cerebrospinal fluid (CSF) biomarker results showing evidence of early AD. Such results consisted of reduced CSF Aβ-42, increased CSF tau, and, using the definition given by the National Institute on Aging at National Institutes of Health and the Alzheimer’s Association (NIA-AA), a diagnosis of mild cognitive impairment (MCI) due to AD (MCI-AD) or mild dementia due to AD. No ongoing drug trials have enrolled patients with advanced AD, which reflects the present consensus that anti-amyloid therapy is not beneficial for patients in the late stage of AD. Compared with 2017 and 2018 (Fig. 2, Table 2), the number of anti-amyloid phase 3 drug trials was lower in 2019, and anti-amyloid trials have also moved to the early stages of AD, including the prodromal or even preclinical stage. AD surrogate biomarkers have been used frequently as secondary outcome measures. The most common outcome biomarkers in trials have been CSF amyloid, CSF tau, volumetric MRI, and amyloid PET [37]. AD Composite Score (ADCOMS), which combines scores on items derived from the AD Assessment Scale–cognitive subscale (ADAS-cog), clinical dementia rating (CDR) score, and Mini-Mental Status Examination (MMSE), has been a useful measure of cognitive outcome in trials concerning early-stage AD with limited cognitive deficits [38].

**Table 2:** Ongoing phase 3 trials on anti-amyloid therapy in AD in 2019

Agent	Mechanism of action	Target type and therapeutic purpose	ClinicalTrials.gov identifier	Status	
Plasma exchange with albumin 1 immunoglobulin	Plasma exchange	Remove amyloid	NCT01561053	Completed	
ALZT-OP1a + ALZT-OP1b	Mast cell stabilizer, anti-inflammatory	Amyloid-related and antineuroinflammatory	NCT02547818	Active, not recruiting	
ANAVEX2–73	Anti-tau, Anti-amyloid	Anti-tau, anti-amyloid, and antineuroinflammatory	NCT03790709	Recruiting	
Crenezumab	Monoclonal antibody directed at oligomers	Remove amyloid	NCT02670083.NCT03114657.NCT03491150	Completed	
E2609 (elenbecestat)	BACE inhibitor	Reduce amyloid production	NCT02956486.NCT03036280	Active, not recruiting	
Gantenerumab	Monoclonal antibody	Remove amyloid	NCT02051608.NCT01224106.NCT03444870.NCT03443973	NCT02051608.NCT01224106 Active, not recruiting, NCT03444870.NCT03443973 Recruiting	
Gantenerumab and Solanezumab	Monoclonal antibody	Remove amyloid/reduce amyloid production	NCT01760005	Recruiting	
GV-971 (sodium oligomannurarate)	Aβ aggregation inhibitor	Amyloid-related	NCT02293915	Completed	
Solanezumab	Monoclonal antibody	Remove amyloid and prevent aggregation	NCT02008357	Active, not recruiting

**Table 3:** Ongoing phase 3 trials on nonanti-amyloid therapy in AD in 2019

Agent	Mechanism of action	Target type and therapeutic purpose	NCT number	Status
AC-1204	Induction of ketosis	Metabolic; symptomatic cognitive enhancer	NCT01741194	Completed
AGB101 (levetiracetam)	SV2A modulator	Amyloid-related and neuroprotective; disease-modifying therapy	NCT03486938	Recruiting
Aripiprazole	Partial agonist at dopamine D2 and 5-HT 1A receptors	Neurotransmitter based; symptomatic cognitive enhancer	NCT02168920	Terminated
AVP-786	Sigma-1 receptor agonist, NMDA receptor antagonist	Neurotransmitter based; BPSD (agitation)	NCT03393520	Recruiting
			NCT02446132	Recruiting
			NCT02442765	Completed
			NCT02442778	Completed
AXS-05	Sigma-1 receptor agonist; NMDA receptor antagonist and dopamine-norepinephrine reuptake inhibitor	Neurotransmitter based; BPSD (agitation)	NCT03226522	Recruiting
Azeliragon	Microglial activation inhibitor, antagonist of the receptor for advanced glycation end products	Amyloid-related and antineuroinflammatory; disease modifying therapy	NCT02916056	Terminated
			NCT02080364	Terminated
			NCT03980730	Recruiting
OPC-34712 (brexpiprazole)	A partial agonist at serotonin 5-hydroxytryptamine1A and dopamine D2 receptors and an antagonist at serotonin 5-hydroxytryptamine2A	Neurotransmitter based; BPSD (agitation)	NCT01922258	Completed
			NCT01862640	Completed
			NCT03620981	Recruiting
			NCT03548584	Recruiting
			NCT03594123	Recruiting
			NCT03724942	Recruiting
Coconut oil	Reduction in ADP-ribosylation factor 1 protein expression	Anti-amyloid, antineuroinflammatory, anti-oxidative, and neuroprotective; symptomatic cognitive enhancer	NCT01883648	Terminated
COR388	Bacterial protease inhibitor	Antineuroinflammatory; disease-modifying therapy	NCT03823404	Recruiting
Escitalopram	Serotonin reuptake inhibition	Neurotransmitter based; BPSD (agitation)	NCT03108846	Recruiting
Gabapentin Enacarbil	Glutamate receptor-independent mechanisms	Neurotransmitter based and neuroprotective; symptomatic cognitive enhancer	NCT03082755	Recruiting
*Ginkgo biloba*	Antioxidant and anti-amyloid aggregation	Antioxidant and anti-amyloid; symptomatic cognitive enhancer	NCT03090516	Recruiting
Guanfacine	Alpha-2A-adrenoceptor agonist, a potent 5-HT2B receptor agonist	Neurotransmitter based; symptomatic cognitive enhancer	NCT03116126	Recruiting
Icosapent ethyl (IPE)	Omega-3 fatty acids protect neurons from disease	Neuroprotective; disease-modifying therapy	NCT02719327	Recruiting
Idalopirdine	5-HT6 receptor antagonist	Neurotransmitter based; symptomatic cognitive enhancer	NCT02006641	Completed
			NCT01955161	Completed
			NCT02006654	Completed
			NCT02079246	Completed
RVT-101 (intepirdine)	5-HT6 receptor antagonist	Neurotransmitter based; symptomatic cognitive enhancer	NCT02586909	Terminated
			NCT02585934	Completed
Insulin (Humulin® R U-100)	Metabolic	Metabolic; symptomatic cognitive enhancer	NCT01767909	Completed
ITI-007 (lumateperone)	A potent 5-HT2A antagonist	Neurotransmitter based; symptomatic cognitive enhancer	NCT02817906	Terminated
Losartan, amlodipine, aerobic exercise training, and others	Angiotensin II receptor blocker, calcium channel blocker, cholesterol agent	Antineuroinflammatory and metabolic; symptomatic cognitive enhancer	NCT02913664	Recruiting
Masitinib	Selective tyrosine kinase inhibitor	Antineuroinflammatory; disease modifying therapy	NCT01872598	Active, not recruiting
Methylphenidate	Dopamine reuptake inhibitor	Neurotransmitter based; BPSD (apathy)	NCT03811847	Recruiting
			NCT02346201	Recruiting
Mirtazapine	Alpha-1 antagonist	Neurotransmitter based; BPSD (agitation)	NCT03031184	Recruiting
MK-4305 (suvorexant)	Orexin antagonist	BPSD (sleep)	NCT02750306	Completed
EVP-6124	Selective α7 nicotinic acetylcholine receptor partial agonist	Cholinergic system; symptomatic cognitive enhancer	NCT02004392	Terminated
			NCT01969136	Terminated
			NCT01969123	Terminated
Nabilone	Agonists at cannabinoid receptors 1 and 2 (CB1/2)	Neurotransmitter based; BPSD (agitation)	NCT02351882	Completed
Nilvadipine	Dihydropyridine calcium channel blocker	Amyloid-related, neuroprotective, and antineuroinflammatory; disease-modifying therapy	NCT02017340	Completed
AVP-923 (nuedexta)	Uncompetitive NMDA glutamate receptor antagonist, a sigma-1 receptor agonist, and a serotonin and norepinephrine reuptake inhibitor	Neurotransmitter based and neuroprotective; symptomatic cognitive enhancer	NCT01832350	Unknown
Pioglitazone	Peroxisome proliferator-activated receptor gamma (PPAR-γ) agonists	Antineuroinflammatory and neuroprotective; symptomatic cognitive enhancers	NCT02284906	Terminated
			NCT01931566	Terminated
Troriluzole	Glutamate modulator	Neuroprotective; disease modifying therapy	NCT03605667	Recruiting
TRx0237 (LMTX)	Tau stabilizers and aggregation inhibitors	Anti-tau; disease-modifying therapy	NCT01689233	Completed
			NCT02245568	Terminated
			NCT03446001	Recruiting
Vitamin D3 (cholecalciferol)	Agonist of vitamin D receptor and other membrane-based receptors such as MARRS	Metabolic; symptomatic cognitive enhancer	NCT01409694	Completed
Zolpidem Zoplicone	Allosteric modulator of GABA-A receptors	Neurotransmitter based; BPSD (sleep)	NCT03075241	Recruiting

BPSD = behavioral psychological symptoms of dementia; NMDA = N-methyl-D-aspartate

AN-1792 is the first active immunotherapy strategy for AD that consists of a synthetic full-length Aβ peptide. In 2002, an AN-1792 trial was terminated. In a phase 2 study, 6% of patients developed aseptic meningoencephalitis as a side effect [39]. In 2019, only one active immunotherapy trial combined CAD106 and CNP520 to treat individuals with the ApoE4 allele and amyloid burden without cognitive impairment. CAD106 combines multiple copies of Aβ1–6 peptide derived from the N-terminal B cell epitope of Aβ, coupled to a Qβ virus-like particle [40]. CNP520 (umibecestat) is an orally ingested, small-molecule inhibitor of aspartyl protease and beta-scretase-1 (BACE-1). It is designed to interfere with the upstream process of the amyloid cascade to inhibit Aβ production. The Alzheimer’s Prevention Initiative Generation Program (Generation Study 1), which consists of a CAD106 injection arm versus a placebo or oral CNP520 (50 mg) arm versus a placebo, has announced that the CNP520 arm showed a worsening of cognitive function. However, the CAD106 treatment arm is ongoing. Bapineuzumab was the first monoclonal antibody used for passive immunotherapy strategy to target Aβ in AD. Further trials were discontinued after the first two trials were completed and yielded no treatment effect on either cognitive or functional outcomes [41]. In 2019, five drug trials were conducted using monoclonal antibody targeting Aβ, namely aducanumab, crenezumab, gantenerumab, and solanezumab, and one trial with a combination of gantenerumab and solanezumab. Aducanumab targets aggregated Aβ forms. In the brain, it preferentially binds to parenchymal over vascular amyloid [42]. Studies have shown that amyloid deposition was reduced in all treatment groups at 26 weeks and further reduced by the end of the first year. Additionally, amyloid was cleared from the six cortical regions of interest, namely the frontal, parietal, lateral temporal, sensorimotor, anterior, and posterior cingulate areas [43]. The most common side effect was amyloid-related imaging abnormalities (ARIA). In ARIA, the white spots in the MRI, which represent vasogenic edema, were mostly found in the ApoE4 carriers and in participants receiving high doses. In 2017 and 2018, the long-term open-label extension phase of the Multiple Dose Study of Aducanumab (BIIB037) (Recombinant, Fully Human Anti-Aβ IgG1 mAb) in Participants With Prodromal or Mild Alzheimer’s Disease (PRIME study), which is a phase 1b study evaluating the safety, tolerability, and pharmacokinetics/pharmacodynamics of aducanumab in patients with prodromal/mild AD aged 50–90 years with positive amyloid PET scan, was reported to be continuing to show dose-dependent amyloid removal and also slowing cognitive decline. However, in March 2019, Biogen and Eisai announced the termination of the phase 3 ENGAGE (221 AD301 Phase 3 Study of Aducanumab (BIIB037) in Early Alzheimer’s Disease) and EMERGE (221 AD302 Phase 3 Study of Aducanumab (BIIB037) in Early Alzheimer’s Disease) trials of aducanumab because a futility analysis concluded that these trials would not reach their primary endpoint—slowing of disease progression as measured by the CDR-Sum of Boxes (CDR-SB). The futility analysis was based on data available as of on December 26, 2018 from 1748 patients. However, additional data from these studies became available thereafter and it resulted in a large dataset consisting of a total of 3285 patients, including 2066 with the full 18 months of treatment. The updated analysis revised the results of EMERGE to be statistically significant, especially for the patients treated with a high dose of aducanumab. Those patients showed a significant reduction in decline of global functions from baseline in CDR-SB scores at 78 weeks (23% versus placebo, *P* = 0.01), the ADAS-Cog 13 (27% versus placebo, *P* = 0.01), and AD Cooperative Study–Activities of Daily Living Inventory, Mild Cognitive Impairment version (40% versus placebo, *P* = 0.001). Imaging of amyloid plaque deposition in EMERGE demonstrated that amyloid plaque burden decreased with low- and high-dose aducanumab compared with placebo at 26 and 78 weeks (*P* < 0.001). The company announced its plan to file a Biologics License Application in early 2020 [44]. Solanezumab is a humanized IgG1 monoclonal antibody that targets the central region of Aβ. In phase 3 trials, the Progress of Mild Alzheimer’s Disease in Participants on Solanezumab Versus Placebo (EXPEDITION) 1, EXPEDITION 2, and EXPEDITION 3 studies had enrolled patients with mild to moderate AD with intravenous solanezumab infusions, which failed to show efficacy with regard to cognitive and functional outcomes. Florbetapir PET analysis did not show a reduction in brain amyloid deposits with solanezumab [45, 46]. Furthermore, solanezumab is being tested in preventive paradigms in the ADCS A4 and DIAN-TU trials (Table 2). Gantenerumab is a completely human recombinant monoclonal IgG1 antibody that binds to both amino-terminal and central regions of Aβ. Gantenerumab shows higher affinities for Aβ oligomers and fibrils than for Aβ monomers [47]. The Marguerite RoAD study evaluated monthly subcutaneous injections of gantenerumab in patients with mild AD. Preliminary results from open-label extension studies indicated that gantenerumab has an acceptable safety profile at a high dose [48]. Furthermore, gantenerumab is being evaluated in the Safety and Efficacy Study of Gantenerumab in Participants With Early Alzheimer’s Disease (GRADUATE) 1, GRADUATE 2, and Dominantly Inherited Alzheimer Network Trials Unit (DIAN-TU) trials (Table 2). Crenezumab is a humanized anti-Aβ monoclonal IgG4 with particular affinity for all pentameric, oligomeric and fibrillary amyloid [49]. Crenezumab is being evaluated in the CREAD (A Study of Crenezumab Versus Placebo to Evaluate the Efficacy and Safety in Participants With Prodromal to Mild Alzheimer’s Disease) trials concerning prodromal to mild AD (Table 2). E2609 (elenbecestat) is a BACE-1 inhibitor. A phase 2b study on elenbecestat in amyloid-PET-positive patients with MCI, prodromal AD, or mild AD showed decreased CSF Aβ levels in a dose-dependent manner but no significant improvements in the Alzheimer’s Disease Composite Score or CDR-SB score [50, 51]. The efficacy of elenbecestat is being evaluated in the A 24-Month Study to Evaluate the Efficacy and Safety of Elenbecestat in Subjects With Early Alzheimer’s Disease (MISSION AD1) and MISSION AD2 trials concerning prodromal AD. These trials will continue until December 2023. GV-971 (sodium oligo-mannurarate) can bind to multiple sites of amyloid, further destabilize and inhibit Aβ aggregation, and then increases Aβ clearance [52]. GV-971 also can reshape gut microbiota and suppress dysbiosis-induced neuroinflammation [53]. A phase 3 study, which began in April 2014, investigated the effects of GV-971 in mild to moderate AD. The primary endpoint is the change in ADAS-Cog 12 score. Reports from this trial showed that GV-971 provides significant cognitive benefits. On November 2, 2019, Shanghai Green Valley Pharmaceuticals announced that China National Medical Product Administration (NMPA) had conditionally approved GV-971 for the treatment of mild to moderate AD [54]. Nilvadipine is a blocker of the dihydropyridine calcium channel. The functions of neuroprotection and anti-inflammation of nilvadipine may contribute to the reduction of Aβ production and the enhancement of Aβ clearance [55].

In 2013, the NILVAD trial measured the efficacy of nilvadipine in people with mild to moderate AD. The primary endpoint was ADAS-Cog. The report revealed no change in primary or secondary outcome measures [56].

Although amyloid plaques are regarded as a pathological hallmark of AD, the causal relationship between amyloid deposition and neurodegeneration was unclear for a long time. Aβ has widespread distribution through the brain and body, even in cognitively normal individuals. Soluble Aβ exerts a physiological function, modulating synaptic function and facilitating neuronal growth; furthermore, Aβ protects the brain from infections, repairs leaks in the blood–brain barrier, and promotes recovery from injury [57, 58]. A study involving cognitively healthy adults showed that Aβ in CSF or the hippocampus increases after sleep deprivation or slow-wave sleep disruption, which indicates the complexity of Aβ kinetics [59, 60, 61]. Disease-modifying agents for chronic conditions such as AD should be started as early as possible in the course of the pathophysiology. This might be the key lesson of the prior large-scale anti-amyloid trials. Researchers now focus on the prodromal or preclinical stage of AD, because Aβ deposition could occur decades earlier than the clinical symptoms of AD manifest. Trials target the stages of even mild cognitive decline may be too late because the brain has been damaged by Aβ and some irreversible processes have been initiated. Several BACE inhibitor trials have reported that even participants who receive treatment have worse cognitive functions (Table 1). Moreover, the evidence from a failure of reversal of cognitive declines in amyloid-targeting drug trials supports this assumption; for example, patients with AD in whose brains Aβ plaques were virtually cleared by anti-amyloid immunotherapy did not show cognitive benefit [62]. However, the newly released results from the EMERGE trial indicate that decreasing amyloid load in the brain is beneficial, which suggests that aducanumab could modify, but not reverse, the disease course, thus slowing cognitive decline. Moreover, the amyloid hypothesis has evolved [9, 10, 14, 63]. One concept is that Aβ oligomers might impair neuronal function by causing synaptic dysfunction, inducing mitochondrial dysregulation and affecting microglia [64]. The other lesson of the prior large-scale anti-amyloid trials is the need for further basic research regarding metabolism, molecular structures, immune responses, and amyloid toxicity.

### Antineuroinflammation therapy

Azeliragon is an antagonist of the receptor for advanced glycation end products (RAGE). RAGE regulates multiple physiological effects, including the transport of circulating plasma Aβ to the brain, inflammatory process, oxidation stress, and cerebral blood flow [65]. Two phase 3 clinical trials, a 2-year extension study of azeliragon for patients with AD (Evaluation of the Efficacy and Safety of Azeliragon (TTP488) in Patients With Mild Alzheimer’s Disease, STEADFAST Extension) and a study to evaluate the efficacy and safety of azeliragon for patients with mild AD, were discontinued because those trials failed to achieve their primary endpoints in June 2018. The ongoing phase 3 trial to test the effect of azeliragon on patients with mild AD and impaired glucose tolerance started on June 27, 2019. It is scheduled to end in July 2023.

AD-4833 (Pioglitazone) is an insulin sensitizer for peroxisome-proliferator-activated receptor gamma (PPARγ) agonists. It binds to PPARγ to regulate the metabolism of glucose and lipid, and it also mediates the response of microglia to increase Aβ phagocytosis and decrease cytokine release, neuroinflammation, and Aβ levels [66]. A phase 3 clinical trial to qualify the biomarkers for MCI-AD risk and for evaluating the efficacy of pioglitazone in delaying its onset was started in August 2013. The primary outcome measures included the difference of time to diagnosis of MCI-AD for placebo-treated and pioglitazone-treated participants in the low-risk and high-risk groups. The study was terminated because of lack of efficacy in September 2018. Another phase 3 clinical trial was started in February 2015 to investigate the effect of pioglitazone in high-risk participants with cognitive decline and who had completed the TOMORROW (Biomarker Qualification for Risk of Mild Cognitive Impairment (MCI) Due to Alzheimer’s Disease (AD) and Safety and Efficacy Evaluation of Pioglitazone in Delaying Its Onset) study with an adjudicated diagnosis of MCI-AD. The primary outcome measures included the change in the composite score of a broad cognitive test battery. The trial was discontinued in May 2018 because of a lack of efficacy.

### Anti-tau therapy

TRx0237 (LMTX) is a tau aggregation inhibitor. It decreases the level of aggregated tau proteins to alleviate tau-related neuronal damage [67]. A TRx0237 trial exploring the efficacy of TRx0237 in mild AD was initiated in October 2012 and ended in May 2016. The primary outcome measures for the clinical trial were changes in the performance of two scales, the ADAS-cog 11 and the ADCS-ADL 23. The report of this trial revealed that TRx0237 failed to be an add-on treatment for AD [68]. In August 2014, a phase 3 trial was started to evaluate the effect of LMTX in AD or behavioral-variant frontotemporal dementia. It was discontinued in May 2017, and the reason for termination has not been disclosed. The ongoing phase 3 trial on TRx0237 began in January 2018. This trial is intended to compare the efficacy of TRx0237 in different doses in participants with early AD. The primary endpoint is the change in the standardized uptake value ratio based on temporal lobe 18F-fluorodeoxyglucose PET. This trial is scheduled to continue until December 2020.

AADvac1 is an active vaccine that induces the immune response by targeting multiple key epitopes in pathological forms of tau, thereby inhibiting tau aggregation and decreasing the formation of neurofibrillary tangles [69, 70]. A phase 2 trial of AADvac1 was started in March 2016 and was scheduled to continue until June 2019. The purpose of this study was to evaluate the safety and efficacy of 24 months of AADvac1 treatment in patients with mild AD. Primary outcome measures were the safety and tolerability of AADvac1 based on adverse effects, vital signs, electrocardiogram, laboratory data, brain MRI, physical and neurological examination, the Columbia suicide severity rating scale, and a review of the patient diary. The trial progress is still unclear.

Zagotenemab (LY3303560) is a passive immunotherapy. It is an anti-tau antibody engineered to capture and neutralize tau aggregate [71]. A phase 1 trial to investigate the safety of LY3303560 in participants with mild AD began in January 2017. The primary endpoint was the number of participants with serious adverse events. The trial was completed on June 5, 2019, but the report has not been provided. An ongoing phase 2 trial of LY3303560 is evaluating the safety and effectiveness of the treatment in patients with early symptomatic AD. The primary outcome measure is the change in the integrated AD rating scale. The trial is still active with an estimated completion date of August 2021.

### Neuroprotection

BHV-4157 (troriluzole) is a glutamate modulator. Glutamatergic deregulation may lead to brain cell death or dysfunction through destruction of synaptic function and plasticity, promotion of microglia-mediated neuroinflammation, and the release of Aβ and tau [72]. Through increasing the expression of glutamate transporters, BHV-4157 can reduce synaptic glutamate level and increase the synaptic glutamate absorption. In July 2018, phase 2 and 3 trials were initiated to evaluate the efficacy of BHV-4157 in patients with mild to moderate AD. The primary outcome measure is the change in ADAS-Cog 11. The trials are scheduled to continue until February 2020.

Coconut oil comprises medium-chain fatty acids (MCFAs) with a high amount of medium-chain triglycerides. Coconut oil downregulates the expression of ADP-ribosylation factor 1, thereby inhibiting the secretion and aggregation of Aβ and restraining the expression of APP [73]. MCFAs could be converted into ketone bodies, which are related to the improvement of mitochondrial function and reduction of oxidation [74]. Coconut oil can resist oxidation and neuroprotection. A phase 3 clinical trial to investigate the effect of coconut oil in mild to moderate AD was initiated in June 2013. However, it was terminated in February 2017. The reasons for the termination were funding limitations and a low enrollment rate.

*Ginkgo biloba* extrat (GBE) might improve cognitive function through multiple mechanisms, including regulating kinase signaling pathways, enhancing vasodilation, affecting neurotransmitter levels, ameliorating cerebrovascular circulation, and neuroplasticity [75]. It blocks certain functions of platelet-activating factor, leading to the inhibition of platelet aggregation, suppression of neuroinflammation, and prevention of cell damage caused by free radicals [75, 76]. Phase 2 and 3 trials to investigate the efficacy of GBE in the treatment of mild to moderate AD began in August 2016. The primary outcomes include changes in the MMSE, ADAS-cog, activities of daily life scale, neuropsychiatric inventory, geriatric depression scale, electroencephalography P300, renal function, liver function, and 1.5 T MRI. The trials are scheduled to continue until March 2020.

### Cognitive enhancers

RVT-101 (intepirdine) is a postsynaptic 5-hydroxytryptamine (5-HT) 6 receptor antagonist. The antagonist mediates the balance between excitatory and inhibitory signals through the regulation of GABA and glutamate levels in different neuronal circuits. Moreover, it increases the release of several neurotransmitters, including dopamine, norepinephrine (NE), and ACh [77]. The phase 3 MINDSET clinical trial investigated the effect of intepirdine in patients with mild to moderate AD receiving donepezil 5 or 10 mg daily. The MINDSET trial was started in October 2015 and was completed in September 2017. The primary outcome measures included changes in the scales of ADAS-cog 11 and ADCS-ADL 23. This study failed to achieve its primary endpoints. However, a statistically significant result in a secondary outcome, an improvement in the clinician interview-based impression of change plus caregiver interview, was observed. A phase 3 MINDSET extension trial was started in April 2016. It investigated the safety of RVT-101 for participants with AD who had completed the RVT-101-3001 study. The primary endpoints included the occurrence of adverse events and changes in physical examinations, vital signs, electrocardiograms, and routine laboratory assessments. The trial was terminated in March 2018 because it did not reach the primary endpoints in study RVT-101-3001.

EVP-6124 is an α7 nicotinic acetylcholine receptor agonist and a 5-HT3 receptor antagonist and mediates the release of multiple neurotransmitters, such as γ-aminobutyric acid, glutamate, ACh, and dopamine [78, 79]. It improves cognitive performance by enhancing cholinergic neurotransmission. In October 2013, two phase 3 trials enrolled patients with mild to moderate AD taking an AChEI currently or previously in different countries. The primary outcomes included changes in ADAS-Cog 13 and CDR-SB. In June 2014, a phase 3 trial was started to evaluate the safety of EVP-6124 in patients with AD who completed study EVP-6124-024 or EVP-6124-025. In September 2015, the FDA issued a clinical hold on these three AD studies due to a gastrointestinal adverse effect. The clinical hold on these trials continues.

### BPSD-relieving therapy

AXS-05 is a combination of dextromethorphan (DMP) and bupropion. DMP is an N-methyl-D-aspartate (NMDA) receptor antagonist, a glutamate receptor modulator, a sigma-1 receptor agonist, and an inhibitor of the serotonin and NE transporters. Bupropion is a dopamine-NE reuptake inhibitor and CYP2D6 inhibitor, increasing the pharmacodynamics of DMP [80]. Excessive activity of the NMDA receptor is toxic to cells and accelerates cell death [81]. An ongoing phase 3 trial is investigating the efficacy of AXS-05 on agitation in patients with AD. The primary endpoint is the change in the Cohen-Mansfield Agitation Inventory (CMAI) score.

ITI-007 (lumateperone) is a multitarget-directed ligand. It is a 5-HT2A receptor antagonist, a serotonin reuptake inhibitor, a glutamate GluN2B receptor phosphoprotein modulator, and a presynaptic agonism and postsynaptic antagonism at D2 receptors. It regulates the release, uptake, and delivery of a variety of neurotransmitters [82]. A phase 3 trial to evaluate the efficacy of ITI-007 in patients with AD experiencing agitation was initiated in June 2016. The primary outcome measure was the change in CMAI-C. The trial was terminated in December 2018 because it failed to meet its primary endpoint after analyzing prespecified interim data.

Apiprazole is a dopamine D2 agonist, a 5-HT receptor 1A receptor agonist, and a 5-HT2A antagonist, acting as dopamine system stabilizers (DSSs). DSSs reduce dopaminergic neurotransmission when excessive dopamine activity in the mesocortical pathway occurs. Thus, DSSs decrease the hyperactivity of dopaminergic neurons, which mediates psychosis, and DSSs restore the dopamine activity in the cortical regions that are thought to be related to cognitive impairment [83]. Furthermore, aripiprazole improves the Aβ-induced reduction of neurite outgrowth. This therapy potentially overcomes the neurotoxicity caused by Aβ in AD-related psychosis [84]. A phase 3 clinical trial compared the efficacy of different dosages of aripiprazole in patients with AD experiencing agitation. The primary outcome endpoint was change in the CMAI. The trial was terminated in March 2016 because it was difficult to enroll participants.

MK-4305 (suvorexant) is a dual antagonist of orexin receptors [85]. The diurnal variation of orexin neuronal activity regulates the circadian rhythm. The orexin system regulates the sleep–wake state due to its role in accelerating and maintaining wakefulness and arousal [85, 86]. A phase 3 trial investigated the efficacy of suvorexant in patients with mild to moderate AD experiencing insomnia. The trial started in May 2016 and was completed in September 2018. The primary outcome measures included changes in polysomnography (derived total sleep time), prevalence of adverse events, and withdrawal rate caused by adverse events. Merck announced that the results of this trial met the primary and secondary endpoints. Suvorexant is a promising candidate in the treatment of behavioral and psychological symptoms in AD-related dementia.

## Conclusions

Effective or disease-modifying drugs for AD are still lacking. The molecular and clinical events, including amyloid accumulation, neuroinflammation, tau accumulation, neural degeneration, cognitive decline, and occurrence of behavioral psychological symptoms, develop along with AD progression. The clinical trials targeting these events are under evaluation. Because the trials of anti-amyloid failed in recent years, the research focus has shifted to populations at prodromal or preclinical stages with positive diagnostic biomarkers. Meanwhile, the amyloid hypothesis has been challenged, and the number of anti-amyloid phase 3 trials was reduced significantly in 2019. The targets of phase 1 and 2 trials are diverse, and the trends show increased targeting of neuroprotection and antineuroinflammation in phase 1 and phase 2 trials, respectively. Chronic progressive disorders usually require two or more drugs to effectively slow down the disease progression. Prospectively, it may be reasonable to conduct trials with “dirty drugs” which have actions at multiple targets, namely anti-amyloid and anti-tau effects, neurotransmitter modification, anti-neuroinflammatory and neuroprotective effects, and cognitive enhancement.

## Data Availability

Data sharing is not applicable to this article because no datasets were generated or analyzed during the current study.
